# Endotrophin and C6Ma3, serological biomarkers of type VI collagen remodelling, reflect endoscopic and clinical disease activity in IBD

**DOI:** 10.1038/s41598-021-94321-2

**Published:** 2021-07-19

**Authors:** Majken Lindholm, Line E. Godskesen, Tina Manon-Jensen, Jens Kjeldsen, Aleksander Krag, Morten A. Karsdal, Joachim H. Mortensen

**Affiliations:** 1 Biomarkers and Research, Nordic Bioscience, Herlev Hovedgade 205-207, 2730 Herlev, Denmark; 2 Department of Medical Gastroenterology, University of Southern Denmark and Odense University Hospital, Odense, Denmark

**Keywords:** Biomarkers, Inflammatory bowel disease

## Abstract

In inflammatory bowel disease (IBD), the chronic inflammation deeply affects the intestinal extracellular matrix. The aim of this study was to investigate if remodeling of the intestinal basement membrane type VI collagen was associated with pathophysiological changes in Crohn’s disease (CD) and ulcerative colitis (UC). Serum from IBD patients (CD: n = 65; UC: n = 107; irritable bowel syndrome: n = 18; healthy subjects: n = 20) was investigated in this study. The serological biomarkers C6Ma3 (a matrix metalloproteinase (MMP) generated fragment of the type VI collagen α3 chain) and PRO-C6, also called endotrophin (the C-terminus of the released C5 domain of the type VI collagen α3 chain) were measured by ELISAs. Serum C6Ma3 was increased in CD patients with moderate to severe and mild endoscopically active disease compared to endoscopic remission (p = 0.002, p = 0.0048), respectively, and could distinguish endoscopically active disease from remission with an AUC of 1.0 (sensitivity: 100%, specificity: 100%) (p < 0.0001), which was superior to CRP. C6Ma3 was increased in CD patients with moderate to severe clinical disease compared to mild and remission (p = 0.04; p = 0.009). Serum PRO-C6, endotrophin, was increased in CD patients in clinically remission compared to mild disease (p = 0.04) and moderate to severe disease (p = 0.065). In UC, fecal calprotectin was the only marker that alone could distinguish both clinical and endoscopic active and inactive disease. Type VI collagen degradation of the α3 chain mediated by MMPs was increased in CD patients with endoscopically active disease, measured by the serological biomarker C6Ma3, which was able to distinguish endoscopically active from inactive CD.

## Introduction

Inflammatory bowel disease (IBD) patients, being Crohn's disease (CD) and ulcerative colitis (UC) patients, experience intestinal tissue damage during their disease course^1^. The chronic inflammation, which is most severe in CD, profoundly affects the intestinal extracellular matrix (ECM), which may manifest as excessive ulcers, intestinal stenosis, or fistulas.

In IBD, disease activity assessment is primarily based on clinical symptoms and endoscopy with support from biochemical markers. The most used biochemical biomarkers for disease activity assessment are C-reactive protein (CRP) and fecal calprotectin (FC)^2,3^. Evidence suggest that mucosal healing predicts long term remission, reduced number of hospitalization, and improved disease control^4,5^ and since mucosal healing has emerged as the treatment goal in IBD, these biochemical biomarkers have been correlated to endoscopic disease activity^6^. Furthermore, the scoring indexes used for endoscopic disease assessment have been evaluated and modified to improve the description of intestinal tissue inflammation and damage^7^. However, there is still no objective and generally accepted definition of mucosal healing^8^, and since it is documented by endoscopy, which is invasive, time-consuming, and unpleasant for the patients, non-invasive biomarkers would be valuable in disease monitoring. Biomarkers of the ECM may serve as objective measures of tissue damage and potentially, mucosal healing^1^.

ECM remodeling is a normal physiological process, but during inflammation, the remodeling is altered, which is predominantly due to a change in the expression and activation of matrix metalloproteinases^1,9,10^. As a result of remodeling, fragments of the ECM can be measured in the circulation of IBD patients. Increased remodeling of type I, III, V, VI collagen has already been reported^11–15^, and degradation of type VI collagen in CD patients was associated with CRP > 5 mg/L^14^. Furthermore, type VI collagen is increased in the intestinal tissue of IBD patients, with a marked upregulation in stenotic areas^16^.

Type VI collagen is highly abundant in the basement membrane of the intestinal epithelial cells (IECs) at the entire crypt-villus axis, but also around smooth muscle cells, blood vessels^17,18^, and in abdominal adipose tissue^19^. Type VI collagen is a regulator of the fibronectin fibrillogenesis and affects IEC morphology and possibly affects their behavior by regulating cell-fibronectin interactions^18^. The type VI collagen monomer is composed of three alpha chains (α1, α2, α3)^20^, with the α3 chain being the largest^21,22^. The monomers assemble into dimers and tetramers intracellularly and are then shed into the extracellular space where they form a characteristic beaded microfilament structure^23–26^. Due to the variety of globular domains at the N- and C-terminus of especially the α3 chain, type VI collagen interacts with a variety of ECM proteins^27–30^, and cells^28,31^. The C5 domain of the α3 chain is cleaved off immediately after secretion into the extracellular space^32^. This domain is called endotrophin and can enhance angiogenesis, fibrosis, inflammation, and promote tumor progression^33^. Therefore, type VI collagen has both structurally and cell-regulating properties^26,28^. ECM degradation, including type VI collagen, is involved in the pathology in UC patients^34^; still, the influence of type VI collagen in intestinal mucosal damage and healing is unknown.

Both PRO-C6 and C6Ma3 are biomarkers of the type VI collagen α3 chain. PRO-C6 reflects the formation of the α3 chain, and C6Ma3 reflects the subsequent degradation of the α3 chain^35,36^. Thus, the two biomarkers measure different pathophysiological processes of the same protein. The serological biomarker C6Ma3, an MMP-2 and MMP-9 generated fragment of the α3 chain of type VI collagen, a neo-epitope, has been shown to be elevated in IBD patients and different types of cancers, including colorectal cancer^36,37^. The serological biomarker of type VI collagen formation, PRO-C6 is the C-terminus of the released C5 domain of type VI collagen α3 chain and also called endotrophin, which is associated with severity of a variety of diseases^38–42^. Thus, it is evident that type VI collagen remodeling is an important pathological feature in various diseases including IBD. But the relevance of type VI collagen remodeling in IBD needs to be validated and further elucidated.

The aim of this study was to investigate if type VI collagen remodeling was associated with pathophysiological changes in subgroups of IBD patients. The primary objective was to determine if serum levels of C6Ma3 and PRO-C6 were related to endoscopic disease activity.

## Materials and methods

### Study subjects

The study subjects were enrolled in a prospective observational study performed at Odense University Hospital (ClinicalTrials.gov ID: NCT02612103). The study subjects were CD (n = 65), UC (n = 107), irritable bowel syndrome (IBS) patients (n = 18) and healthy subjects (n = 20). The data reported in this paper are specifically focused on baseline data on the CD and UC patients with the focus on ECM biomarkers in relation to clinical and endoscopic disease activity. The subject characteristics are presented in Table 1. Before sample collection, informed signed consent was collected from each study subject. The study was approved by the Regional Ethics Committee of Southern Denmark (journal number: S-20150107) and conducted according to the Declaration of Helsinki.

**Table 1:** Subject characteristics.

	CD n = 65	UC n = 107	IBS n = 18	HS n = 20
**Age**				
Mean (range)	36.32 [18–76]	45.20 [18–80]	39.0 [21–71]	40.6 [19–68]
**Sex**				
Males (%)	36 [55.4]	62 [57.9]	13 [72.2]	12 [60]
Females (%)	29 [44.6]	45 [42.1]	5 [27.8]	8 [40]
**Smoking**				
Current (%)	19 [29]	9 [8]	2 [11]	1 [5]
Former (%)	17 [26]	48 [45]	3 [17]	5 [25]
Never (%)	28 [43]	50 [47]	13 [72]	14 [70]
BMI	25 [16–51]	26 [17–56]	24 [19–37]	26 [19–44]
Disease duration	6.7 [0–38]	8.3 [0.1–39]		
**Treatment**^**a**^				
5-ASA, topical	0 [0]	30 [28]		
5-ASA, oral	7 [11]	71 [66]		
Corticosteroids, topical	0 [0]	2 [2]		
Corticosteroids, oral	4 [6]	7 [6]		
Corticosteroid, iv	2 [3]	3 [3]		
Immunosuppressant	23 [35]	18 [17]		
Biologic agents	39 [60]	22 [21]		
No treatment	6 [9]	12 [11]		
**Clinical disease activity**				
HBI (remission/mild/moderate/severe) (n = 65)	41/13/10/1			
Mayo partial (remission/mild/moderate/severe) (n = 107)		52/23/28/4		
**Endoscopic disease activity**				
SES-CD (remission/mild/moderate/severe) (n = 17)	7/3/5/2			
Mayo endo (remission/mild/moderate/severe) (n = 63)		11/21/20/11		
**Montreal classification CD**				
Age at diagnosis (A1/A2/A3) (n = 65)	10/46/9			
Location (L1/L2/L3/L4) (n = 65)	10/24/31/0			
Behavior (B1/B2/B3) (n = 65)	38/19/8			
**Montreal classification UC**				
Disease extension (E1/E2/E3) (n = 107)		34/47/26		

HBI: remission (< 5), mild (5–7), moderate (8–16), severe (> 16). Mayo partial: remission (< 2), mild (2–4), moderate (5–7), severe (> 7). SES-CD: Simple endoscopic score of Crohn’s disease: remission (0–2), mild (3–6), moderate (7–15), severe (> 15). Mayo endo: remission (0), mild (1), moderate (2), severe (3). A1: below 16 years, A2: between 17 and 40 years, A3, above 40 years. L1: ileal, L2: colonic, L3: ileocolonic, L4: isolate upper disease. B1: non-stricturing, non-penetrating, B2: stricturing, B3: penetrating. E1: proctitis, E2: Left sided, E3: extensive.

*CD* Crohn’s disease, *UC* ulcerative colitis, *IBS* irritable bowel syndrome, *HS* healthy subjects.

^a^Some patients received more than one treatment.

### Data collection

The clinical data, blood samples, stool samples, and endoscopies were collected and performed by gastroenterologists at Odense University Hospital, Denmark. The assessment of clinical disease activity was recorded using the Harvey-Bradshaw Index (HBI) (0–4: remission; 5–7: mild activity; 8–16: moderate activity; > 16: severe activity) and the partial Mayo score (< 2: remission; 2–4: mild activity; 5–7: moderate activity; > 7: severe activity). The IBD patients were classified according to the Montreal classification. The blood samples were used for a variety of biochemical analyses carried out at Odense University Hospital. The endoscopic score for disease activity was recorded for some of the IBD patients. The Simple Endoscopic Score for CD (SES-CD)^43^ (0–2: remission; 3–6: mild disease activity; 7–15: moderate disease activity, > 16: severe disease activity) and Mayo endoscopic subscore^44^ for UC (0: inactive disease; 1: mild activity, 2: moderate activity, 3: severe activity) were applied.

### Biomarker measurements

The serological biomarkers, C6Ma3^36^ and PRO-C6^35^, were measured at Nordic Bioscience (Herlev, Denmark). The biomarkers were measured using solid-phase competitive enzyme-linked immunosorbent assays (ELISA). A 100 µL solution of biotinylated antigen in assay buffer was added to 96-well streptavidin-coated plates (Roche Diagnostics cat: 11940279) and incubated for 30 min at 20 °C while shaking at 300 rpm. The standard peptide, kit controls, and serum samples were added to the wells. For the C6Ma3 assay, a 100 µL solution of the primary antibody, specific for the C6Ma3 neo-epitope, was added, and the solution was incubated for 1 h at 20 °C while shaking at 300 rpm. Afterward, a 100 µL solution of the secondary antibody was added to the wells and incubated for 1 h at 20 °C while shaking at 300 rpm. For the PRO-C6 assay, a solution of horse-radish peroxidase-conjugated antibody specific for the PRO-C6 neo-epitope was added to the wells and incubated at 4 °C for 20 h while shaking 300 rpm. After each incubation, the plates were washed five times with washing buffer. One hundred µL of tetramethylbenzidine (TMB) was added to each well and incubated for 15 min in darkness at 20 °C while shaking at 300 rpm. The reaction was ended by adding 100 µL of stop solution (0.1 M H_2_SO_4_) to each well. The absorbances were read at 450 nm with 650 nm as reference using a VersaMax ELISA microplate reader (Molecular Devices LLC). The standard curve was plotted using a four-parameter mathematical fit model.

### Statistical analysis

Ordinary one-way ANOVA with Tukey's test for multiple comparisons and Kruskal–Wallis with Dunn's test for multiple comparisons were used to test the difference in biomarker levels between disease groups, between patients with different clinical disease activity based on the HBI and partial Mayo scores, and between patients with different endoscopic disease activity based on SES-CD and Mayo endoscopic scores. Due to the small sample size, the CD groups with severe clinical (HBI > 16) and severe endoscopic (SES-CD > 15) disease activity were merged with their respective moderate disease activity. Spearman's rank and Pearson correlations were used to test the biomarkers' correlation to clinical and endoscopic disease activity. To investigate the biomarkers' ability to distinguish clinically active from inactive CD and UC patients, we grouped the CD patients based on HBI: remission (0–4) and active disease (> 5), and the UC patients with the partial Mayo score: remission (0–1) and active disease (≥ 2). To investigate the biomarkers' ability to identify patients with endoscopically active disease, patients with endoscopically active disease (SES-CD > 2; Mayo endo > 0) were grouped and compared to patients in endoscopic remission (SES-CD = 0–2; Mayo endo = 0). Unpaired t-tests and Mann–Whitney tests were used to test the difference between biomarker levels in patients that were either in remission or had active disease, and the area under the ROC curve analysis was used to test the biomarkers ability to distinguish these groups. Youden's J index was used to determine the cut-off in the ROC curve analyses. Spearman r correlations were used to identify potential confounding factors and correlations between the ECM biomarkers and CRP and FC. Logistic regression models were used to test and adjust for the potential confounding factors (age, BMI, gender, smoking). Least squared multiple regression models were applied to investigate the effect of combining the biomarkers into one model. p-values < 0.05 were considered statistically significant. GraphPad Prism 8.3.0 and MedCalc were used for the statistical analyses. Due to the insufficient amount of serum for some of the study subjects, there were a few missing values for some of the biomarkers, which was reported in Table 5.

## Results

### Biomarker levels in disease groups and healthy subjects

The serum C6Ma3 was significantly elevated in healthy subjects compared to CD, UC, and IBS patients (p = 0.0122, p = 0.0102, p = 0.0326) (Table 2). FC was significantly elevated in both CD and UC patients compared to IBS patients (p < 0.0001, < 0.0001) (Table 2). PRO-C6 and CRP were not significantly different between the four groups (Table 2). In CD patients C6Ma3 levels were correlated to CRP (r = 0.532, p < 0.0001) and FC (r = 0.292, p = 0.035), while in UC patient C6Ma3 were correlated to CRP (0.275, p = 0.006), but not to FC (r = 0.15, p = 0.15). In CD and UC patients, PRO-C6 was not correlated no neither CRP (r = − 0.023, p = 0.86; r = 0.126, p = 0.1995) or FC (r = 0.22, p = 0.95; r = 0.098, p = 0.332), respectively (Table 3).

**Table 2:** Biomarker levels.

	CD (n = 65*)	UC (n = 107*)	IBS (n = 18*)	HS (n = 20*)	p-value
C6Ma3 (ng/mL) [range]	2.150 [1.022–5.314] n = 59	2.2 [0.806–5.030] n = 100	2.07 [0.708–3.93] n = 18	3.178 [1.672–9.314] n = 16	CD vs. UC: > 0.9999
					CD vs. IBS: > 0.9999
					CD vs. HS: 0.0122
					UC vs. IBS: > 0.9999
					UC vs. HS: 0.0102
					IBS vs. HS: 0.0326
PRO-C6 (ng/mL) [range]	7.012 [3.632–23-5] n = 65	6.811 [3.902–19.43] n = 106	6.59 [4.28–32.83] n = 18	7.00 [5.264–17.45] n = 20	CD vs. UC: > 0.9999
					CD vs. IBS: > 0.9999
					CD vs. HS: > 0.9999
					UC vs. IBS: > 0.9999
					UC vs. HS: > 0.9999
					IBS vs. HS: > 0.9999
CRP(mg/L) [range]	2.35 [0.3–45] n = 64	2.5 [0.4–91] n = 107	1.55 [0.6–16] n = 18	1.0 [0.6–24] n = 19	CD vs. UC: > 0.9999
					CD vs. IBS: 0.3365
					CD vs. HS: 0.1174
					UC vs. IBS: 0.7164
					UC vs. HS: 0.2756
					IBS vs. HS: > 0.9999
FC (μg/g) [range]	127 [5–4624] n = 58	141 [5–6000] n = 100	15 [5–69] n = 18	–	CD vs. UC: > 0.9999
					CD vs. IBS: < 0.0001
					CD vs. HS: n.a.
					UC vs. IBS: < 0.0001
					UC vs. HS: n.a.
					IBS vs. HS: n.a.

Median values with ranges.

*Changes in sample size due to missing values for the specific biochemical markers. The samples size for each biomarker is stated in the table. Differences in biomarker levels were investigated with Kruskal–Wallis. p-values adjusted with Dunn’s multiple comparisons test are presented in the table. Statistical significance was defined as p < 0.05.

**Table 3:** Correlations between CRP and FC and and the biomarkers C6Ma3 and PRO-C6.

	CD				UC			
	C6MA3		PRO-C6		C6Ma3		PROC-6	
CRP	r = 0.532^a^	p < 0.001	r = − 0.023^a^	p = 0.86	r = 0.275^a^	p = 0.006	r = 0.126^a^	p = 0.1995
FC	r = 0.292^a^	p = 0.035	r = 0.22^a^	p = 0.95	r = 0.15^a^	p = 0.15	r = 0.098^a^	p = 0.332

^a^Spearman r; Statistical significance was defined as p < 0.05.

### The serological biomarkers of type VI collagen degradation and formation, C6Ma3 and PRO-C6, were associated with clinical disease activity in CD

**Figure 1:**
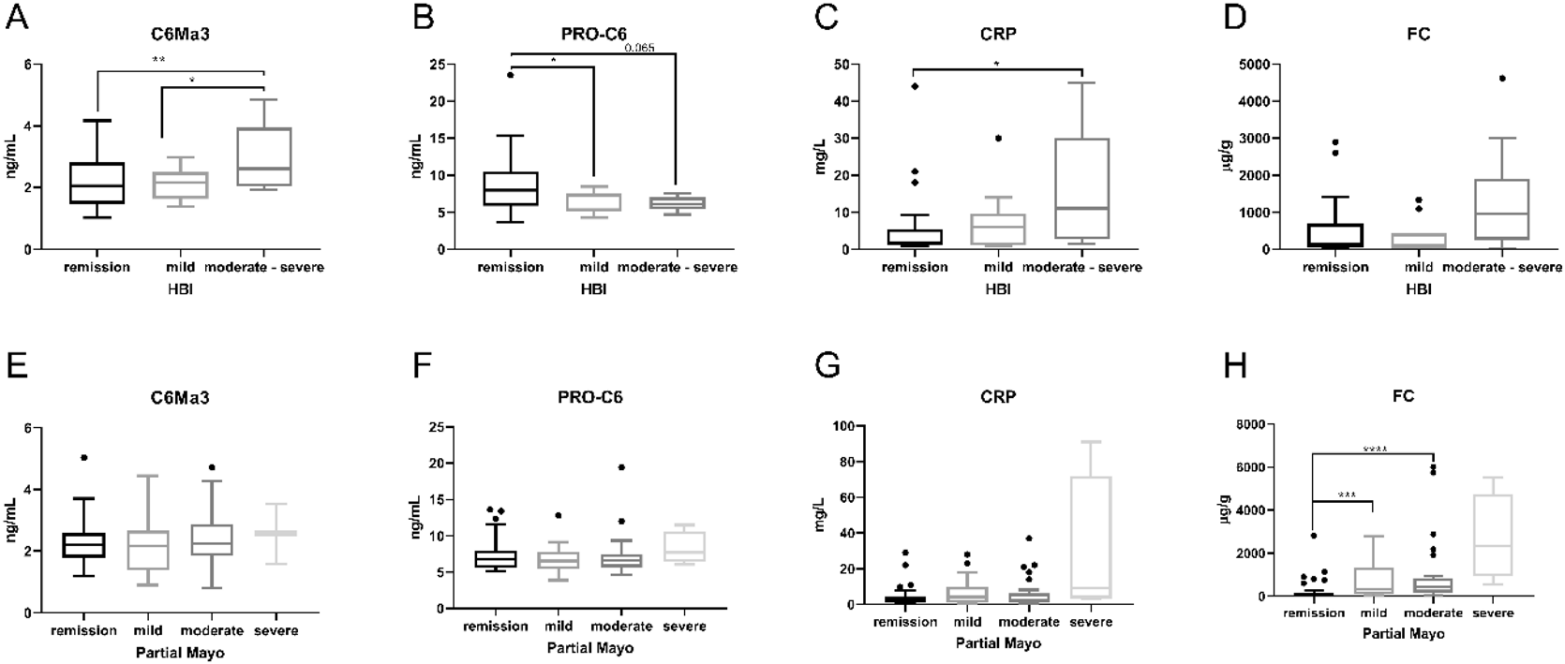
Levels of the biomarkers C6Ma3 (**A**, **E**), PRO-C6 (**B**, **F**), CRP (**C**, **G**), and FC (**D**, **H**) in CD and UC patients grouped by the clinical scoring indexes HBI and Mayo partial. Ordinary one-way ANOVA with Tukey’s multiple comparisons test and Kruskal–Wallis tests with Dunn’s test for multiple comparison were used were applied. *p < 0.05, **p < 0.01, ***p < 0.001, ****p < 0.0001.

CD patients with clinically moderate to severe disease activity had significantly increased serum C6Ma3 compared to patients with clinically mild disease activity or in clinical remission (p = 0.04; p = 0.009) (Fig. 1A). In contrast, serum PRO-C6 was significantly increased in CD patients in clinically remission compared to CD patient with clinically mild activity (p = 0.04) and borderline significantly elevated in CD patients in remission compared to patients with moderate to severe disease activity (p = 0.065) (Fig. 1B). CRP levels were significantly increased in CD patients with clinically moderate to severe activity compared to patients in remission (p = 0.013), while FC was not significantly elevated in patients with clinically active disease (Fig. 1C,D). C6Ma3, CRP, FC demonstrated moderate positive correlations to HBI (p = 0.01, p = 0.001, p = 0.008) (Table 4). PRO-C6 demonstrated a trend of weak negative correlation to HBI (Table 4). In addition, a least-squares multiple regression model of serum C6Ma3 and PRO-C6, including the common confounding factors (age, BMI, gender, and smoking), improved the correlation of C6Ma3 to the HBI score (spearman r = 0.4666, p = 0.0003), but PRO-C6 did not improve this correlation alone (Table 4). Serum PRO-C6 was able to distinguish clinically active from inactive CD patients with an AUC of 0.73 (sensitivity: 100%, specificity: 46%) (p = 0.0002) (Table 5), while serum C6Ma3 demonstrated an AUC of 0.65 (sensitivity: 89%, specificity: 47%) (p = 0.053) (Table 5). Combining C6Ma3 and PRO-C6 with the common confounding factors in a logistic regression model improved the discriminative power to an AUC of 0.86 (sensitivity: 100%, specificity: 55%) (p < 0.0001) (Table 5). CRP could distinguish clinically active form inactive CD patients with an AUC of 0.68 (sensitivity: 63%, specificity: 75%) (p = 0.01), while FC did not distinguish these two groups (Table 5).

**Table 4:** Correlations between biomarkers and clinical and endoscopic disease activity scores.

	CD				UC			
	HBI		SES-CD		Mayo partial		Mayo endo	
	r^a,b^	p-value	r^a,b^	p-value	r^a,b^	p-value	r^a,b^	p-value
C6Ma3	0.3250^a^	0.0128	0.689^a^	0.0042	0.09175^a^	0.3640	0.2201^a^	0.0968
PRO-C6	− 0.2308^a^	0.0643	− 0.1399^a^	0.5896	− 0.0367^a^	0.7091	0.03593^a^	0.7816
CRP	0.3940^a^	0.0013	0.4716^a^	0.0577	0.1808^a^	0.0624	0.1972^a^	0.1214
FC	0.3428^a^	0.0084	0.7117^a^	0.0019	0.5978^a^	< 0.0001	0.6457^a^	< 0.0001
C6Ma3 + PRO-C6^c^	0.3295^b^	0.0123	0.4571^b^	0.075	0.0937^a^	0.3668	0.2210^a^	0.0985
C6Ma3 + PRO-C6 + confounding factors^c^	0.4666^b^	0.0003	0.7711^b^	0.0005	0.1694^a^	0.0938	0.4252^a^	0.001

Correlations between clinical (HBI and Mayo partial) and endoscopic scores (SES-CD and Mayo endo) and the biomarkers C6Ma3, PRO-C6, CRP and FC.

^a^Spearman r; ^b^pearson r. Statistical significance was defined as p < 0.05.

^c^Least squares multiple regression model. Confounding factors: age, BMI, gender, and smoking.

**Table 5:** AUROC analysis of the biomarkers in endoscopically active vs endoscopically inactive CD and UC patients.

	CD patients: endoscopically remission vs endoscopically active		UC patients: endoscopically remission vs endoscopically active	
	AUC [95% CI] [sensitivity; specificity]	p-value	AUC [95% CI] [sensitivity; specificity]	p-value
C6Ma3	1.00 [0.79–1.00] [100-100]	< 0.0001	0.57 [0.38–0.76] [71-45]	0.49
PRO-C6	0.59 [0.33–0.81] [50-86]	0.56	0.52 [0.34–0.69] [71-36]	0.86
CRP	0.87 [0.62–0.98] [91-71]	< 0.0001	0.52 [0.32–0.72] [50-64]	0.81
FC	0.81 [0.60–1.09] [60-100]	0.004	0.85 [0.74–0.95] [69-90]	< 0.001
C6Ma3 + PRO-C6^a^	1.00 [0.79–1.00] [100-100]	< 0.0001	0.59 [0.45–0.71] [85-36]	0.41
C6Ma3 + PRO-C6 + confounding factors^a^	1.00 [0.79–1.00] [100-100]	< 0.0001	0.66 [0.53–0.78] [67-73]	0.14

	CD patients: clinically remission vs clinically active		UC patients: clinically remission vs clinically active	
	AUC [95% CI] [sensitivity; specificity]	p-value	AUC [95% CI] [sensitivity; specificity]	p-value
C6Ma3	0.65 [0.51–0.77] [89-47]	0.053	0.53 [0.53–0.63] [45-71]	0.589
PRO-C6	0.73 [0.60–0.83] [100-46]	0.0002	0.54 [0.44–0.64] [17-96]	0.481
CRP	0.684 [0.56–0.79] [63-75]	0.013	0.59 [0.49–0.68] [38-82]	0.106
FC	0.62 [0.48–0.74] [52-78]	0.147	0.82 [0.73–0.89] [92-70]	< 0.0001
C6Ma3 + PRO-C6^a^	0.76 [0.63–0.87] [89-58]	< 0.0001	0.57 [0.46–0.67] [33-86]	0.258
C6Ma3 + PRO-C6 + confounding factors^a^	0.86 [0.74–0.94] [100-55]	< 0.0001	0.641 [0.54–0.74] [52-76]	0.012

*AUROC* area under the receiver operating characteristic curve, *AUC* area under the curve, *CI* confidence interval.

^a^Logistic regression model. Confounding factors: age, BMI, gender, and smoking.

### Increased serum level of the type VI collagen degradation biomarker, C6Ma3, associated with endoscopic disease activity in CD patients

**Figure 2:**
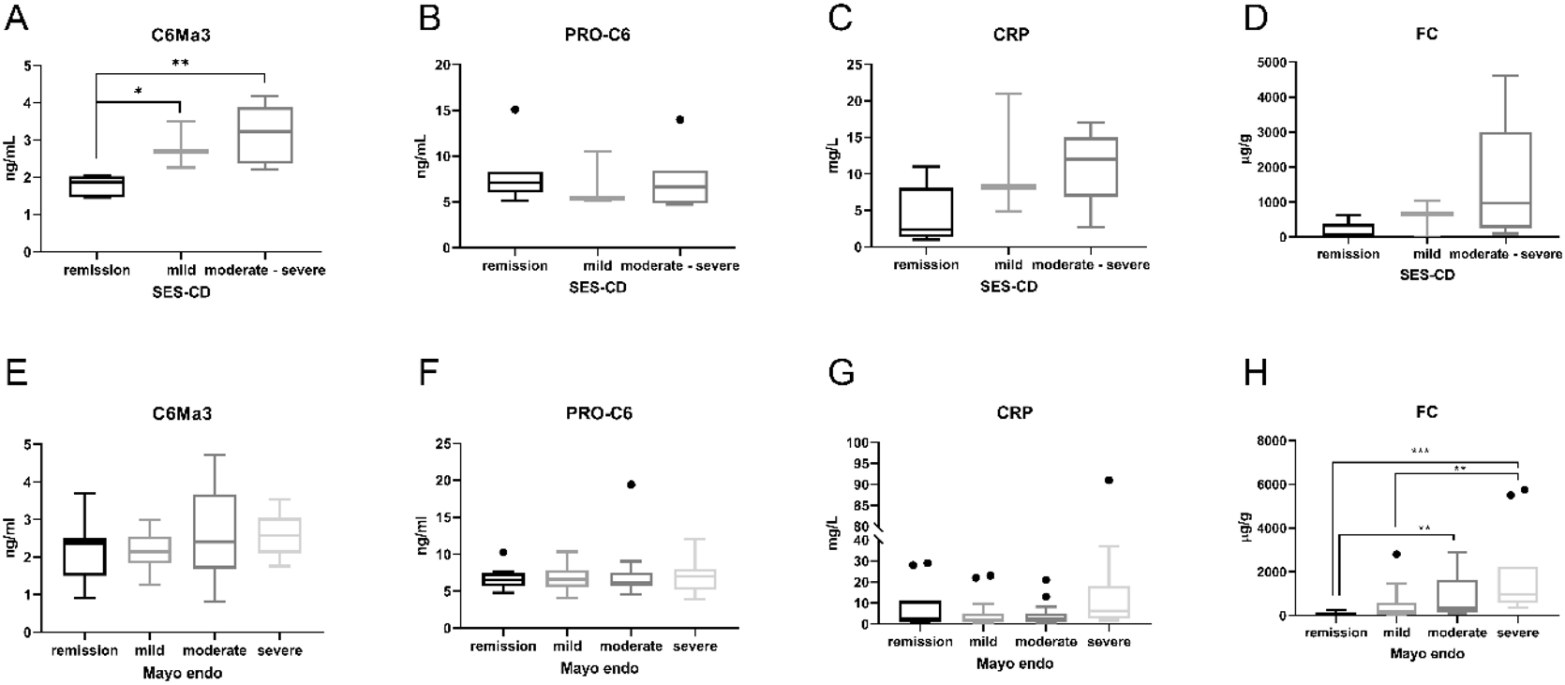
Levels of the biomarkers C6Ma3 (**A**, **E**), PRO-C6 (**B**, **F**), CRP (**C**, **G**), and FC (**D**, **H**) in CD and UC patients grouped by the endoscopic scoring indexes SES-CD and Mayo endo. Ordinary one-way ANOVA with Tukey’s multiple comparisons tests and Kruskal–Wallis with Dunn’s test for multiple comparison were used to test for difference in biomarker levels between groups. *p < 0.05, **p < 0.01, ***p < 0.001.

**Figure 3:**
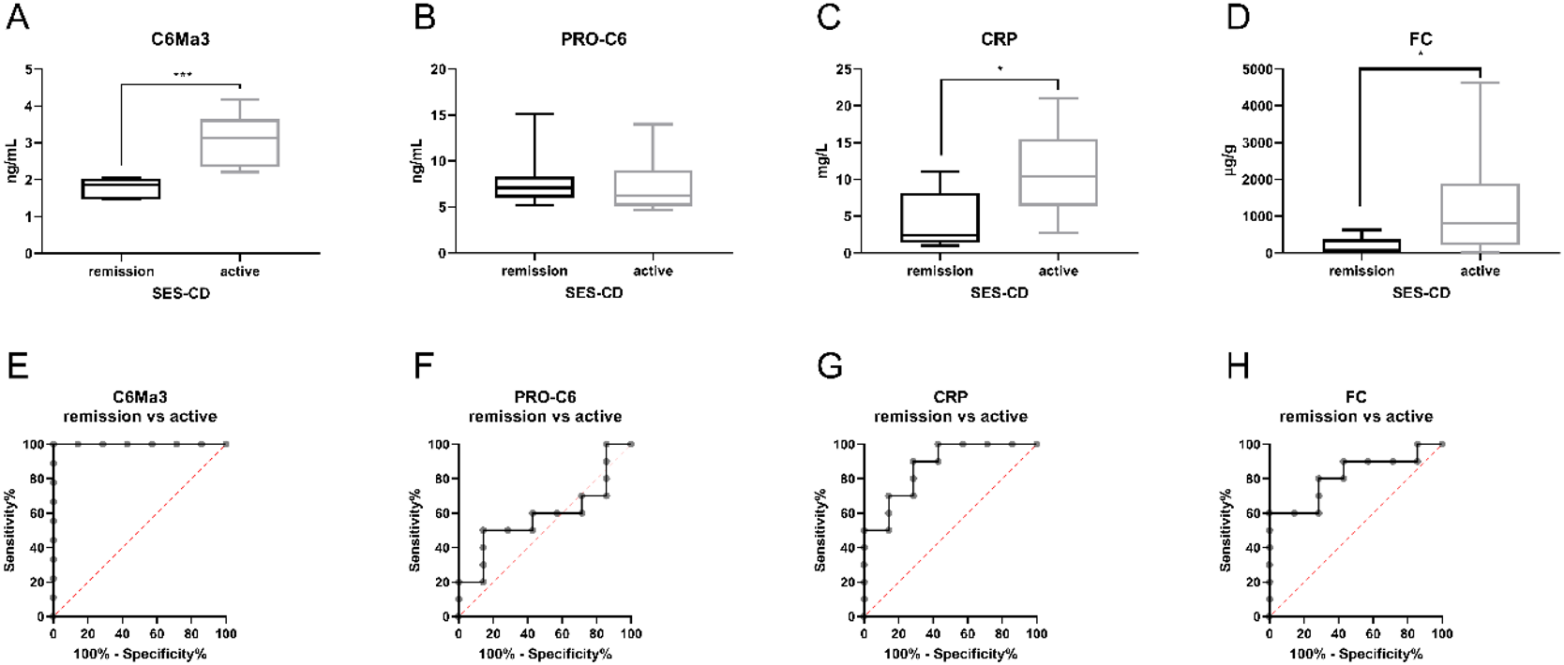
Levels and respective ROC-curves of the biomarkers C6Ma3 (**A**, **E**), PRO-C6 (**B**, **F**), CRP (**C**, **G**), and FC (**D**, **H**) in CD patients grouped in endoscopically remission (SES-CD ≤ 2) or active (SES-CD > 2). Unpaired tests and Mann Whitney test were used to test for difference in biomarker levels. *p < 0.05, ***p < 0.001.

Serum C6Ma3 was strongly correlated to SES-CD (spearman r = 0.689, p = 0.004) in CD patients (Table 4) and CD patients with moderate to severe and mild endoscopically active disease had significantly higher C6Ma3 levels than CD patients in endoscopically remission (p = 0.002, p = 0.0048), which was not observed for PRO-C6, CRP, and FC (Fig. 2A–D). A least-squares multiple regression model of serum C6Ma3, PRO-C6, and the common confounding factors (age, BMI, gender, and smoking) improved the correlation to SES-CD (spearman r = 0.77, p = 0.0005). A model of only C6Ma3 and PRO-C6 reduced the correlation compared to serum C6Ma3 alone, and serum PRO-C6 alone was not correlated to SES-CD. FC was strongly correlated to SES-CD, while CRP was only borderline significantly correlated to SES-CD (Table 4). C6Ma3, CRP, and FC were significantly elevated in serum from CD patients with endoscopically active disease (p < 0.001, p = 0.015, p = 0.03), whereas PRO-C6 was not increased (Fig. 3A–D). C6Ma3 was the most powerful biomarker to distinguish endoscopically active disease from endoscopically remission in CD patients with an AUC of 1.00 (sensitivity: 100%, specificity: 100%) (p < 0.0001) (Fig. 3E). CRP and FC distinguished endoscopically active CD patients from inactive patients with an AUC of 0.87 (sensitivity: 90%, specificity: 71%) (p < 0.0001) and 0.81 (sensitivity: 60%, specificity: 100%) (p = 0.004), respectively (Fig. 3G,H). Serum PRO-C6 did not distinguish endoscopically active from inactive CD patients (Fig. 3F). Comparing CD patients with different disease behaviors and locations based on the Montreal classification did not show and difference in the biomarker levels of C6Ma3 nor PRO-C6 (data not shown).

### Biomarkers of type VI collagen remodeling were not associated with clinical nor endoscopic disease activity in UC patients

UC patients with clinically active disease did not have increased C6Ma3 and CRP serum levels nor lower levels of PRO-C6 as CD patients (Fig. 1E–G). However, FC were significantly increased in UC patients with clinically moderate and mild disease activity compared to patients in remission (p < 0.0001, p = 0.0007) (Fig. 1H) and demonstrated a strong significant correlation to the partial Mayo score (Spearman r = 0.5978, p < 0.0001) (Table 4). Also, C6Ma3, PRO-C6, and CRP were not associated with active endoscopic disease in UC patients, which however was observed for FC (Fig. 2E–H). A least-squares multiple regression model of serum C6Ma3 and PRO-C6, including the common confounding factors, did not correlate to the partial Mayo score (Table 4). A logistic regression model of C6Ma3, PRO-C6 and the common confounding factors could distinguish UC patients with clinically active disease from inactive disease with an AUC of 0.64 (sensitivity: 52%, specificity: 76%) (p = 0.012). FC could distinguish clinically active from inactive UC patients (AUC of 0.82 (sensitivity: 92%, specificity: 70%) (p < 0.0001)) (Table 5). A least-squares multiple regression model of C6Ma3, PRO-C6 and the common confounding factors had a positive correlation to the Mayo endoscopic score (spearman r = 0.4252, p = 0.001), which was an improvement of serum C6Ma3 alone (spearman r = 0.2201, p = 0.0968). But a model of only C6Ma3 and PRO-C6 alone did not improve the C6Ma3 correlation to the Mayo endoscopic score (Table 4). PRO-6 and CRP did not correlate to Mayo endoscopic score, but FC had a strong positive correlation to the Mayo endoscopic score (spearman r = 0.6457, p < 0.0001) (Table 4). Only FC performed well in distinguishing endoscopically active UC patients from the patients in endoscopically remission, with an AUC of 0.85 (sensitivity: 69%, specificity: 90%) (p < 0.001) (Table 5).

## Discussion

Chronic intestinal inflammation drives protease-mediated ECM remodeling and causes tissue damage in IBD^9^. The aim of this study was to investigate if type VI collagen remodeling was associated with pathophysiological changes in the CD and UC patients with the primary objective to determine if serum levels of C6Ma3 and PRO-C6 were related to endoscopic disease activity in IBD patients. Here, we identified an altered type VI collagen remodeling in IBD patients with the serological biomarkers C6Ma3 and PRO-C6, and the remodeling was different between endoscopically active disease and remission. Even though the levels were lower in patients compared to healthy subjects, sequential measurements of C6Ma3 and PRO-C6 may enlighten the pathophysiological changes in IBD, in particular CD, and help to address endoscopic changes.

Serum C6Ma3, the degradation marker, was elevated in CD patients with clinically moderate disease activity and with moderate to severe endoscopic disease activity. Interestingly, endotrophin, measured with PRO-C6, the formation marker, was not associated with endoscopic disease activity. Still, the degradation of the type VI collagen α-chain, being C6Ma3, was a strong marker for endoscopic disease activity in CD as it could separate endoscopically active and inactive CD patients with 100% sensitivity and 100% specificity. In other diseases, such as chronic kidney disease^38–40^, idiopathic pulmonary fibrosis^41^, and cardiovascular diseases^42^, endotrophin, measured by PRO-C6, was a predictor of disease severity and progression, and type VI formation is, in general, related to adverse disease events^10^. Fibroblasts are the main drivers of type VI collagen formation, while other cell types such as macrophages are responsible for the subsequent degradation of the same protein^10^. Our results show that biomarkers derived from the same protein reflect different pathophysiological processes, and in this study, data suggest that type VI collagen degradation, opposed to its formation, is associated with severe disease.

Still, serum C6Ma3 was superior to CRP and FC in terms of distinguishing endoscopically active CD patients form inactive. Usually, CRP and FC correlate well with endoscopic disease activity in IBD^45–47^, which was also the case in this study, with FC being superior to CRP in UC patients. Due to the nature of CD, endoscopy can be more challenging compared to UC patients. Thus, serological biomarkers, such as C6Ma3, that reflect endoscopic disease activity could be of importance in patients for which endoscopy is difficult to perform and where other biochemical markers are unclear. Since C6Ma3 separates the endoscopically active and inactive CD patients so well, it suggests that this biomarker potentially can reflect mucosal healing as a direct measure of tissue remodeling, which could be valuable in understanding the underlying pathophysiology of CD. The importance of type VI collagen in IBD in relation to fibrosis has recently been reported^48^. Our study supports the relevance of type VI collagen in IBD, and especially in relation to endoscopic disease activity. Furthermore, the positive correlations between C6Ma3 and CRP and FC in both CD and UC patients support the association between active inflammation and increased type VI collagen degradation.

It has been shown that type VI collagen affects IEC behavior through regulation of fibronectin^18^; thus, the BM type VI collagen is essential for IEC homeostasis. Our study supports this notion, as degradation of type VI collagen, shown with the biomarker C6Ma3, is associated with endoscopic disease activity in CD patients. However, C6Ma3 does not show the same trend in UC patients, for which the biomarker was not significantly elevated in patients with endoscopically active disease. This could be due to the difference in endoscopic scoring systems in UC and CD, where the SES-CD includes the amount of intestinal tissue that is inflamed^43^, opposed to the Mayo endoscopic subscore, which does not take the amount/area of tissue involved into account^44^. A biomarker that is a direct measure of tissue remodeling would be expected to correlate better with an endoscopic score that takes tissue involvement into account and, therefore, could explain the difference. However, serum C6Ma3 was elevated in CD patients with clinically moderate active disease, but it was not elevated in UC patients with clinically active disease. This could suggest that type VI collagen remodeling is different in UC and CD, and that the difference is not only related to the endoscopic scoring indexes. An explanation for the difference could be that CD and UC are diseases with different mucosal immune responses, which results in different tissue remodeling driven by MMPs and tissue inhibitors of metalloproteinases (TIMPs), which may explain why C6Ma3 is not related to active disease in UC as CD. Also, previous studies that use ECM remodeling markers of other ECM proteins and neo-epitopes have shown that ECM remodeling differs between CD and UC^12,13^. The fact that the nature of inflammation in CD is transmural, while it is restricted to the mucosa in UC patients, possibly contributes to the difference observed in type VI collagen remodeling due to the difference in ECM involvement. The location of type VI collagen around smooth muscle cells and blood vessels^17^ and its abundance in adipose tissue^19^ may also be a contributor to why type VI collagen remodeling is significant in CD. In addition, one could speculate that creeping fat, which sheets the inflamed intestinal segments and is associated with transmural inflammation and often occurs in CD^49^, may also contribute to an alteration in type VI collagen remodeling with its high content of type VI collagen^19^.

The serum levels of the type VI collagen formation biomarker, PRO-C6/endotrophin, were increased in CD patients with clinically inactive disease compared to clinically active CD patients, which suggests that the formation of type VI collagen is reduced in active CD. This is opposite of C6Ma3, which may be related to different pathophysiological changes that are essential and specific for intestinal inflammation and wound healing. In skin wounds, type VI collagen is initially deposited at the wound edge flowed by increased deposition within the re-epithelialized wound^50^. It is possible, the increased PRO-C6 in CD patients in remission reflects increased deposition of type VI collagen following successful wound healing similar to the mechanism in skin wounds. A combination of decreased type VI collagen formation with the increased degradation of type VI collagen measured by C6Ma3, our data suggest that type VI collagen is predominantly degraded in active CD. Unfortunately, a great proportion of the CD patients with clinically inactive CD did not have an endoscopy performed; thus, it is unknown if the CD patients with high PRO-C6 serum levels with clinically inactive disease also have endoscopically inactive disease. Differences could also reflect the ongoing medical therapy in the patients. The number of patients did not allow us to study this.

We found that by combining C6Ma3 and PRO-C6 in multiple regression models and in logistic regression models in combination with the common confounding factors, the correlation to the HBI and SES-CD improved in CD patients. Also, the ability to distinguish clinically active from inactive CD patients was increased by combining the biomarkers and taking age, BMI, gender, and smoking into account. The additive effect of combining the biomarkers was demonstrated in UC patients, where the correlation to Mayo endoscopic score greatly improved. Furthermore, this model that combines the biomarkers in UC patients demonstrated a significant difference between patients with endoscopic moderate and severe disease activity compared to patients with mild endoscopic disease activity or in remission (data not shown). Also, a composite model of C6Ma3, PRO-C6, and the common confounding factors increased the ability to distinguish clinically active and inactive UC patients. These results suggest that one or more serological biomarkers of ECM remodeling can provide an objective measure of clinical and endoscopic disease activity in IBD.

There are some methodological considerations to this study. The patients were from a well-characterized cohort. Unfortunately, the number of CD patients with active disease and who had an endoscopy performed was rather low. It would be interesting to investigate the type VI remodeling in a larger cohort of CD patients. The patients received different medical therapies, which may influence the biomarker levels. The healthy subjects included in this study had higher levels of C6Ma3 than the disease groups; they also had higher levels than healthy subjects in other studies reporting on this biomarker^36,37^. These studies demonstrated healthy subjects’ levels significantly lower that CD and UC patients with a mean value approximately 0.7 ng/mL^36^, which was 3.7 ng/mL in the current study. Two of the healthy subjects in the current study had CRP values above 5 mg/L and were not excluded from analysis. Exclusion of these two healthy subjects would not significantly change the mean value of C6Ma3, thus they cannot alone explain the high levels of healthy subjects. It is possible handling of the healthy subject samples has been systematically different as compared to the patients. The high levels of C6Ma3 may therefore be due to technical reasons that cannot be accounted for. C6Ma3 levels in healthy subjects in the two other studies of IBD and cancers^36,37^, which were measured with the same lot of the C6Ma3 biomarker, support this notion of a technical matter. Since the markers included in this study were not aimed for a diagnostic purpose, but for disease activity monitoring purposes, it is still relevant to assess the levels of C6Ma3 in relation to disease activity within IBD subgroups.

The median age of UC patients was higher than the age of CD patients (data not shown), and serum C6Ma3 correlated weakly to age and gender in UC patients, and BMI was weakly correlated to PRO-C6. In CD patients, PRO-C6 was weakly correlated to smoking. Logistic regression analysis did not find a marked effect of these potential confounders on the serum levels of C6Ma3 and PRO-C6 in relation to identifying endoscopic and clinical disease activity. The C6Ma3 levels of IBS patients were interestingly within the range of CD, but still there was a significant difference in FC levels. The remodeling of type VI collagen in IBS patients has not been intensely investigated, but this study appoints to a potential similarity in the underlying tissue pathology. Earlier studies have shown that IBS patients and CD patient have some similarities in their tissue remodeling profiles^12^, and further studies need to elucidate on their similarities and differences.

## Conclusion

In summary, we found that type VI collagen degradation of the α3 chain mediated by MMP-2 and MMP-9 was increased in CD patients with endoscopically active disease, measured by the serological biomarker C6Ma3. C6Ma3 was able to distinguish endoscopically active and inactive CD patients with 100% sensitivity and 100% specificity. Due to the association between endoscopic disease activity and C6Ma3 type VI collagen remodeling and biomarkers of this may aid in understanding the underlying tissue pathology in CD and promote a more objective measure of endoscopic and mucosal healing. Interestingly, PRO-C6, a serological biomarker of type VI collagen formation and endotrophin, which is associated with severity in other diseases were not associated with disease activity in this study, which suggest a different role for type VI collagen in the pathophysiological processes in IBD. Finally, type VI collagen remodeling was different in CD and UC as C6Ma3 and PRO-C6 did not seem to be altered in UC patients.

## References

[1] Petrey AC, de la Motte CA (2017). The extracellular matrix in IBD. Curr. Opin. Gastroenterol..

[2] Chang S, Malter L, Hudesman D (2015). Disease monitoring in inflammatory bowel disease. World J. Gastroenterol..

[3] Krzystek-Korpacka M, Kempiński R, Bromke M (2020). Biochemical biomarkers of mucosal healing for inflammatory bowel disease in adults. Diagnostics..

[4] Picco MF, Farraye FA (2019). Targeting mucosal healing in Crohn’s disease. Gastroenterol. Hepatol. (N. Y)..

[5] De Chambrun GP, Peyrin-Biroulet L, Lémann M (2010). Clinical implications of mucosal healing for the management of IBD. Nat. Rev. Gastroenterol. Hepatol..

[6] Langhorst J, Elsenbruch S, Koelzer J (2008). Noninvasive markers in the assessment of intestinal inflammation in inflammatory bowel diseases: Performance of fecal lactoferrin, calprotectin, and PMN-elastase, CRP, and clinical indices. Am. J. Gastroenterol..

[7] Lobatón, T., Bessissow, T., De Hertogh, G., *et al*. The Modified Mayo Endoscopic Score (MMES): A New Index for the Assessment of Extension and Severity of Endoscopic Activity in Ulcerative Colitis Patients. *J. Crohns. Colitis.* 846–852 (2015). 10.1093/ecco-jcc/jjv111 26116558

[8] Klenske, E., Bojarski, C., Waldner, M., *et al*. Targeting mucosal healing in Crohn’s disease: What the clinician needs to know. *Ther Adv Gastroenterol.* **12**, 1–11 (2019). 10.1177/1756284819856865 PMC6572879 31236140

[9] Mortensen JH, Lindholm M, Langholm LL (2019). The intestinal tissue homeostasis—The role of extracellular matrix remodeling in inflammatory bowel disease. Expert Rev. Gastroenterol. Hepatol..

[10] Karsdal, M.A., Nielsen, S.H., Leeming, D.J., *et al*. The good and the bad collagens of fibrosis—their role in signaling and organ function. *Adv. Drug Deliv. Rev.* **121**, 43–56 (2017). 10.1016/j.addr.2017.07.014 28736303

[11] Kjeldsen J, de Muckadell OBS, Junker P (1995). Seromarkers of collagen I and III metabolism in active Crohn’s disease. Relation to disease activity and response to therapy. Gut.

[12] Mortensen JH, Manon-Jensen T, Jensen MD (2017). Ulcerative colitis, Crohn’s disease, and irritable bowel syndrome have different profiles of extracellular matrix turnover, which also reflects disease activity in Crohn’s disease. PLoS ONE.

[13] Mortensen, J.H., Godskesen, L.E., Jensen, M.D., *et al*. Fragments of citrullinated and MMP-degraded vimentin and MMP-degraded type III collagen are novel serological biomarkers to differentiate Crohn’s disease from ulcerative colitis. *J. Crohn’s Colitis*. 1–10. 10.1093/ecco-jcc/jjv123 (2015). 10.1093/ecco-jcc/jjv123 26188349

[14] Van HWT, Mortensen JH, Karsdal MA (2017). Misbalance in type III collagen formation/degradation as a novel serological biomarker for penetrating (Montreal B3) Crohn’s disease. Aliment. Pharmacol. Ther..

[15] Lindholm M, Manon-Jensen T, Madsen GI (2019). Extracellular matrix fragments of the basement membrane and the interstitial matrix are serological markers of intestinal tissue remodeling and disease activity in dextran sulfate sodium colitis. Dig. Dis. Sci..

[16] Scheibe K, Kersten C, Schmied A (2018). Inhibiting Interleukin 36 Receptor Signaling Reduces Fibrosis in Mice with Chronic Intestinal Inflammation.

[17] Groos S, Reale E, Hunefeld G (2003). Changes in epithelial cell turnover and extracellular matrix in human small intestine after TPN. J. Surg. Res..

[18] Groulx JF, Gagné D, Benoit YD (2011). Collagen VI is a basement membrane component that regulates epithelial cell-fibronectin interactions. Matrix Biol..

[19] Pasarica M, Gowronska-Kozak B, Burk D (2009). Adipose tissue collagen VI in obesity. J. Clin. Endocrinol. Metab..

[20] Trüeb B, Winterhalter KH (1986). Type VI collagen is composed of a 200 kd subunit and two 140 kd subunits. EMBO J..

[21] Lamandé SR, Mörgelin M, Adams NE (2006). The C5 domain of the collagen VI α3(VI) chain is critical for extracellular microfibril formation and is present in the extracellular matrix of cultured cells. J. Biol. Chem..

[22] Chu ML, Pan TC, Conway D (1989). Sequence analysis of alpha 1(VI) and alpha 2(VI) chains of human type VI collagen reveals internal triplication of globular domains similar to the A domains of von Willebrand factor and two alpha 2(VI) chain variants that differ in the carboxy terminus. EMBO J..

[23] Furthmayr H, Wiedemann H, Timpl R (1983). Electron-microscopical approach to a structural model of intima collagen. Biochem. J..

[24] Von Der Mark H, Aumailley M, Wick G (1984). Immunochemistry, genuine size and tissue localization of collagen VI. Eur. J. Biochem..

[25] Engvall E, Hessle H, Klier G (1986). Molecular assembly, secretion, and matrix deposition of type VI collagen. J. Cell Biol..

[26] Cescon, M., Gattazzo, F., Chen, P., *et al*. Collagen VI at a glance. *J. Cell Sci.* **128**, 3525–3531 (2015). 10.1242/jcs.169748 26377767

[27] Kuo HJ, Maslen CL, Keene DR (1997). Type VI collagen anchors endothelial basement membranes by interacting with type IV collagen. J. Biol. Chem..

[28] Bonaldo P, Russo V, Bucciotti F (1990). Structural and functional features of the α3 chain indicate a bridging role for chicken collagen VI in connective tissues. Biochemistry.

[29] Bidanset DJ, Guidry C, Rosenberg LC (1992). Binding of the proteoglycan decorin to collagen type VI. J. Biol. Chem..

[30] Wiberg C, Klatt AR, Wagener R (2003). Complexes of matrilin-1 and biglycan or decorin connect collagen VI microfibrils to both collagen II and aggrecan. J. Biol. Chem..

[31] Aumailley M, Mann K, von der Mark H (1989). Cell attachment properties of collagen type VI and arg-gly-asp dependent binding to its α2(VI) and α3(VI) chains. Exp. Cell Res..

[32] Aigner T, Hambach L, Söder S (2002). The C5 domain of Col6a3 is cleaved off from the Col6 fibrils immediately after secretion. Biochem. Biophys. Res. Commun..

[33] Park J, Scherer PE (2012). Adipocyte-derived endotrophin promotes malignant tumor progression. J. Clin. Invest..

[34] Kirov S, Sasson A, Zhang C (2019). Degradation of the extracellular matrix is part of the pathology of ulcerative colitis. Mol. Omi..

[35] Sun S, Henriksen K, Karsdal MA (2015). Collagen type III and VI turnover in response to long-term immobilization. PLoS ONE.

[36] Nielsen, S.H., Mortensen, J.H., Willumsen, N., *et al*. A fragment of collagen type VI alpha-3 chain is elevated in serum from patients with gastrointestinal disorders. *Sci. Rep.* **10**(5910), 1–27 (2020). 10.1038/s41598-020-62474-1 PMC7125205 32245981

[37] Willumsen N, Bager C, Karsdal MA (2019). Matrix metalloprotease generated fragments of type VI collagen have serum biomarker potential in cancer—A proof of concept study. Transl. Oncol..

[38] Stribos EGD, Nielsen SH, Brix S (2017). Non-invasive quantification of collagen turnover in renal transplant recipients. PLoS ONE.

[39] Rasmussen DGK, Hansen TW, Von SBJ (2018). Higher collagen VI formation is associated with all-cause mortality in patients with type 2 diabetes and microalbuminuria. Diabetes Care.

[40] Pilemann-Lyberg S, Rasmussen DGK, Hansen TW (2019). Markers of collagen formation and degradation reflect renal function and predict adverse outcomes in patients with type 1 diabetes. Diabetes Care.

[41] Organ, L., Duggan, A.-M., Oballa, E., *et al*. Biomarkers of collagen synthesis predict progression in the PROFILE idiopathic pulmonary fibrosis cohort. *Respir. Res.* **20**, 148 (2019). 10.1186/s12931-019-1118-7 PMC6624898 31299951

[42] Nielsen SH, Mygind ND, Michelsen MM (2018). Accelerated collagen turnover in women with angina pectoris without obstructive coronary artery disease: An iPOWER substudy. Eur. J. Prev. Cardiol..

[43] Daperno M, D’Haens G, Van AG (2004). Development and validation of a new, simplified endoscopic activity score for Crohn’s disease: The SES-CD. Gastrointest. Endosc..

[44] Schroeder KW, Tremaine WJ, Ilstrup DM (1987). Coated oral 5-aminosalicylic acid therapy for mildly to moderately active ulcerative colitis—A randomized study. N. Engl. J. Med..

[45] Solem CA, Loftus EV, Tremaine WJ (2005). Correlation of C-reactive protein with clinical, endoscopic, histologic, and radiographic activity in inflammatory bowel disease. Inflamm. Bowel Dis..

[46] Saverymuttu SH, Hodgson HJF, Chadwick VS (1986). Differing acute phase responses in Crohn’s disease and ulcerative colitis. Gut.

[47] Reenaers C, Bossuyt P, Hindryckx P (2018). Expert opinion for use of faecal calprotectin in diagnosis and monitoring of inflammatory bowel disease in daily clinical practice. United Eur. Gastroenterol. J..

[48] Scheibe K, Kersten C, Schmied A (2019). Inhibiting interleukin 36 receptor signaling reduces fibrosis in mice with chronic intestinal inflammation. Gastroenterology.

[49] Kredel LI, Siegmund B (2014). Adipose-tissue and intestinal inflammation—Visceral obesity and creeping fat. Front. Immunol..

[50] Theocharidis G, Drymoussi Z, Kao AP (2016). Type VI collagen regulates dermal matrix assembly and fibroblast motility. J. Invest. Dermatol..

